# “To Be Twice as Good to Get Half”: Minorities’ Diminished Returns

**DOI:** 10.31586/jbls.2025.1158

**Published:** 2025-05-05

**Authors:** Shervin Assari, John Ashley Pallera, Hossein Zare

**Affiliations:** 1 Department of Internal Medicine, Charles R. Drew University of Medicine and Science, Los Angeles, CA, United States; 2 Department of Family Medicine, Charles R. Drew University of Medicine and Science, Los Angeles, CA, United States; 3 Department of Urban Public Health, Charles R. Drew University of Medicine and Science, Los Angeles, CA, United States; 4 Marginalization-Related Diminished Returns (MDRs) Center, Los Angeles, CA, United States; 5 College of Medicine, Charles R. Drew University of Medicine and Science, Los Angeles, USA.; 6 Department of Health Policy and Management, Johns Hopkins Bloomberg School of Public Health, Baltimore, MD, United States; 7 School of Business, University of Maryland Global Campus (UMGC), Adelphi, MD, United States

**Keywords:** Minorities’ Diminished Returns, Systemic Barriers, Health Disparities, Socioeconomic Status, Structural Racism, Chronic Stress, Policy Interventions

## Abstract

“To Be Twice as Good to Get Half” is a common mindset among high aspiration and ambition Black individuals in the U.S., capturing the lived reality of Minorities’ Diminished Returns (MDRs). This paper explains that MDRs reflect how, even with high levels of ambition, self-efficacy, education, and income, Black individuals and other marginalized groups do not experience the same protective benefits for health and well-being as White populations. Systemic obstacles embedded within U.S. society weaken the expected returns on socioeconomic achievements for racialized individuals, creating a reality where “being twice as good” still results in lesser outcomes. High-SES Black individuals, for instance, continue to face significant risks for adverse outcomes, such as depression and chronic disease, due to structural inequities across domains like labor market discrimination, segregation, and accumulated disadvantage from childhood. Our analysis identifies key mechanisms—including interpersonal discrimination, lower-quality education, and structural racism in sectors like banking, policing, and real estate—that erode the protective effects of SES across racial lines. Mediating factors, such as chronic stress, allostatic load, and epigenetic changes over the life course, further compound these diminished returns, weakening the expected physical and mental health benefits. Drawing on extensive evidence from U.S. national and local datasets and corroborated by international studies, this paper underscores the necessity of policies that dismantle structural barriers rather than relying solely on SES improvements. Recommendations include implementing multi-sectoral policies, recognizing the unique challenges of middle-class non-White populations, and approaching policy with humility, acknowledging that achieving equity is a long-term endeavor. By challenging the “bootstraps” narrative, this paper advocates for structural interventions aimed at genuine health and economic equity for all racial and socioeconomic groups. While we provide an in-depth analysis of MDRs’ phenomena, mechanisms, mediators, and policy implications, the experience is often distilled as, “I have to be twice as good to get half.”

## 1. Background

The adage “We have to be twice as good to get half” [1] aptly captures the lived experience of highly ambitious Black individuals and other marginalized groups. While ambition and hard work are often celebrated as pathways to success, these qualities are insufficient to guarantee equitable outcomes for racialized individuals [2,3]. For many high-SES Black individuals, achievements in education, employment, and income come at a steep price and do not always translate into the expected benefits, such as better health or enhanced well-being. This reality stems not from personal shortcomings but from deeply embedded structural biases that diminish the rewards of socioeconomic success for minoritized populations. Ambitious Black individuals, for example, may face unique forms of scrutiny from both within and outside their communities—sometimes being labeled as “Oreo” by their peers [4] while remaining underappreciated or discriminated against by the majority [5,6]. These challenges underscore the complex interplay of race, ambition, and societal biases [7], showing that social identity and skin color continue to influence outcomes in ways that ambition alone cannot overcome.

This phenomenon is also evident among high-achieving Black individuals [8], who often remain at risk of adverse health outcomes such as depression and suicide, even when their SES is high. The Minorities’ Diminished Returns (MDRs) framework [9] provides a valuable lens through which to understand these disparities. MDRs suggest that resources like education, income, and self-efficacy yield weaker health and psychological benefits for Black populations than they do for White populations. Socioeconomic success does not serve as a sufficient buffer against the negative effects of structural racism and social inequities, and as a result, Black individuals cannot rely on high SES alone for protection against adverse mental and physical health outcomes. This reality highlights the need to address the systemic factors that limit the benefits of SES for minoritized communities and amplify the health disparities that persist despite individual success.

Self-efficacy, an essential psychological resource that typically correlates with improved outcomes in mortality, education, and employment, exemplifies the diminished returns Black individuals face. For White populations, high self-efficacy is associated with longevity, academic achievement, and career success [10–13]. However, for Black individuals, the protective effects of self-efficacy are weaker. Despite their self-belief and determination, Black individuals encounter structural barriers and discrimination that limit the full potential of their efforts. While self-efficacy may indeed motivate them to pursue ambitious goals, it does not reliably shield them from health risks or provide the same upward mobility experienced by non-Black populations. Interventions focused on fostering self-efficacy among Black youth, while well-intentioned, may thus fall short of expectations if the structural barriers that inhibit the impact of self-efficacy are not addressed.

## 2. Content of This paper

This paper delves into the phenomenon of Minorities’ Diminished Returns (MDRs) [14], which demonstrates that socioeconomic resources—such as education, income, and self-efficacy—yield weaker health and well-being benefits for Black individuals and other marginalized populations than for White individuals. Despite achieving similar or even higher levels of socioeconomic success, these groups do not experience equivalent protective benefits, largely due to systemic barriers embedded within U.S. society. The paper explores various mechanisms driving MDRs [15], including interpersonal and structural discrimination, residential segregation, lower-quality education, and labor market inequities, all of which erode the potential gains of socioeconomic resources for minoritized individuals. Further, it examines how chronic stress, allostatic load, and epigenetic changes mediate these diminished returns, reducing the expected health benefits across the lifespan. Drawing on literature from both U.S. and international studies, this analysis underscores the need for structural interventions that address these systemic barriers. The paper proposes policy recommendations that prioritize dismantling these obstacles to create more equitable health and economic outcomes, with a particular emphasis on multi-sectoral approaches, supporting the non-White middle class, and fostering long-term, humble commitments to equity. In doing so, it challenges the “bootstraps” narrative, advocating for a shift from individual responsibility to structural solutions that ensure everyone can fully benefit from their achievements.

## 3. Minorities Diminished Returns

This pattern of diminished returns is also evident in the relationship between physical health and mental health [16]—a connection that is generally robust but weakened for Black individuals. While good physical health often supports better mental health, this linkage is less pronounced for Black populations, a phenomenon sometimes referred to as the “Black mental health paradox.” [17–21] Researchers have questioned why Black individuals often report relatively stable mental health even in the face of declining physical health, and the answer may lie in the weakened relationship between physical and mental well-being for this population. This disconnect suggests that the systemic pressures and unique social contexts faced by Black individuals may disrupt the expected pathways between health variables, creating a unique health landscape where positive changes in one domain do not necessarily predict improvements in another.

Similarly, the relationship between physical health and happiness is weaker for Black individuals than for White individuals [108]. Health improvements, which are generally expected to lead to greater happiness, do not consistently translate into increased life satisfaction for Black individuals [108]. This disparity further highlights how structural inequities, discrimination, and social stressors inhibit the full benefits of health. The broader social context—marked by systemic racism and marginalization—may dampen the happiness typically associated with good health, revealing a distinct mechanism by which health and happiness are intertwined yet unequally accessible across racial lines [108].

Education and income, two pillars of socioeconomic status, are widely recognized as buffers against psychological distress and promoters of mental health [109]. However, for Black individuals, these resources provide substantially lower mental health benefits than they do for White individuals [60,77]. High education and income levels do not fully protect Black individuals from mental health risks such as anxiety and depression [60,77]. This diminished protective effect is likely exacerbated by the chronic stressors associated with racial discrimination, limited access to quality healthcare, and the challenges of navigating predominantly White professional spaces [5,6,37,66]. These factors can erode the mental health benefits that are generally expected to accompany socioeconomic advancements, further illustrating the limits of SES as a standalone solution for health disparities [110].

Consequently, high-SES Black individuals remain vulnerable to significant mental health risks, including depression and suicide [60,77]. The resilience, self-efficacy, and financial stability that often insulate White individuals from these outcomes are less effective for Black individuals, who continue to encounter systemic obstacles [110]. This enduring vulnerability highlights the limitations of solely enhancing SES as a strategy to close racial health gaps [9]. Instead, policy efforts may acknowledge and address the structural determinants that impede the benefits of socioeconomic resources for minoritized communities [14]. Such an approach requires a paradigm shift toward dismantling systemic barriers, enabling high-SES Black individuals—and marginalized populations broadly—to fully enjoy the benefits of their accomplishments [111–115].

The MDRs framework highlights the limitations of an SES-centric approach to mitigating racial health disparities. Despite achieving significant milestones in education, income, and self-efficacy, high-SES Black individuals often reap only partial benefits from these achievements, remaining vulnerable to adverse outcomes like depression and anxiety. This reality challenges the assumption that economic and educational advancements alone can yield equitable health and well-being. Instead, the MDRs framework reveals the need for policies that go beyond socioeconomic enhancements to address the deeply rooted structural barriers that hinder these gains for minoritized groups. For genuine equity, policies may not only provide opportunities but also dismantle the systems that maintain racialized barriers, ensuring that all individuals can fully benefit from their hard-won resources. The path forward calls for a comprehensive approach—one that recognizes the unique challenges facing high-SES Black individuals and addresses the root causes of their diminished returns.

## 4. Robustness

This framework has been observed across various marginalized groups, including Black [9,22–29], Latino [30–36], Asian [37–40], LGBTQ+ [41,42], immigrant [43–50], and American Indian/Alaska Native (AIAN) [51–53]populations, illustrating a pattern that transcends racial and ethnic lines. Additionally, MDRs are evident across a broad spectrum of outcomes, from physical health to economic stability to life expectancy. The notion of “pulling oneself up by the bootstraps” grossly oversimplifies the complex realities faced by minoritized communities. This myth ignores the systemic barriers that dilute the returns on individual effort and success, particularly for racialized groups. Even when individuals achieve high levels of education, income, or self-efficacy, structural inequities, discrimination, and historical legacies of exclusion persist, undermining the outcomes of their hard work. The pursuit of equity, therefore, cannot rest on individual ambition alone; it requires actively dismantling systemic barriers that continue to limit success for marginalized communities, regardless of their personal achievements.

Numerous large-scale studies have consistently documented the patterns of Minorities’ Diminished Returns (MDRs), illustrating how socioeconomic resources yield weaker health and well-being benefits for minoritized populations. Data from the National Health Interview Survey (NHIS) [54,55] and National Health and Nutrition Examination Survey (NHANES) [56] reveal that despite similar socioeconomic gains, Black and other racialized individuals often experience poorer health outcomes compared to their White counterparts. The Health and Retirement Study (HRS) [57–59] further underscores this pattern in older adults, showing that higher SES does not equally protect Black individuals against age-related health declines. The National Survey of American Life (NSAL) [60], both in its adult and youth components, offers additional evidence, highlighting disparities in mental health returns on SES between Black and White populations. Similarly, both ABCD and the Monitoring the Future study tracks adolescent substance use and demonstrates that SES is less protective for Black youth than for White youth regarding substance use risks [61,62]. The Population Assessment of Tobacco and Health (PATH) study [63,64] and Understanding America Study (UAS) [65] provide further insights into behavioral health, showing that the mental and physical health benefits of higher SES are weaker among racialized groups. Additionally, the Midlife in the United States (MIDUS) study [66] has documented disparities in psychological well-being and physical health among adults, revealing how socioeconomic advancements do not equally translate into mental and physical health benefits across racial lines. Collectively, these studies and many others substantiate the MDRs framework, indicating that structural barriers persistently weaken the relationship between SES and health outcomes for minoritized individuals, underscoring the need for policy responses that go beyond socioeconomic improvements alone.

Another layer of evidence supporting Minorities’ Diminished Returns (MDRs) is the consistency of these findings across various geographic contexts and study designs. While national datasets [67] illustrate these patterns on a broad scale, similar results emerge in more localized studies across diverse urban centers, including Michigan [68], Los Angeles [69], and Baltimore [70]. These city-specific studies reinforce that diminished returns on SES for minoritized populations are not artifacts of sampling frames or methodological biases but represent robust, persistent patterns. The replication of MDRs across both national and local levels, spanning different population demographics and settings, highlights the structural and universal nature of these disparities, underscoring that the weakened benefits of SES for racialized groups are deeply embedded in systemic inequities rather than confined to any particular locale or sample composition. This geographic and methodological robustness further emphasizes the urgency for targeted policies that address these structural barriers to achieve genuine equity.

The patterns of Minorities’ Diminished Returns (MDRs) persist across all age groups, beginning at birth and accumulating throughout the lifespan. Low birth weight (LBW) in newborns, for instance, is more prevalent among high-SES Black women than their White counterparts, highlighting that socioeconomic advantages do not equally translate into improved birth outcomes for minoritized populations [71]. This disparity continues into childhood, adolescence, adulthood, middle age, and even older adulthood, with Black individuals consistently experiencing weaker health, mental health, and economic benefits from SES compared to White individuals at each life stage. The persistence of these diminished returns from birth onward suggests that there is no single “magic window” after birth for intervention to close these gaps. Rather, it underscores that disparities begin early and become compounded over time, making it critical to implement interventions as early as possible. However, even interventions at birth may already be “late,” as structural inequities affecting prenatal health and maternal outcomes have already taken root. This cumulative pattern emphasizes the need for comprehensive and early interventions that address structural inequities across the entire life course to prevent the entrenchment of disparities over time.

Finally, the Minorities’ Diminished Returns (MDRs) framework extends beyond U.S. borders, with international studies consistently revealing similar patterns across diverse social contexts. Research conducted in countries such as Germany, Israel, Mexico, and various European nations [72] demonstrates that the weaker returns on socioeconomic resources for minoritized populations are not unique to the U.S. but are instead a global phenomenon. Although the sources and shapes of marginalization vary—ranging from ethnic and racial discrimination to immigrant status and caste systems—these studies converge on a common finding: marginalized groups, despite achieving higher socioeconomic status, do not experience the same health, mental health, or economic benefits as their majority counterparts. This global evidence underscores that MDRs are rooted in structural inequities that transcend national boundaries, shaped by each country’s unique forms of exclusion and stratification. The cross-national consistency of MDRs highlights the resilience of these disparities, pointing to a need for policies worldwide that recognize and address the specific sources of marginalization in each context, moving toward a more equitable distribution of socioeconomic benefits for all.

## A. Multi-level Causes

### A1. Interpersonal Discrimination

Interpersonal discrimination is a significant mechanism that drives Minorities’ Diminished Returns (MDRs), limiting the full benefits of socioeconomic achievements for marginalized individuals [6,66,73–79]. Daily encounters with discrimination—whether subtle microaggressions or overt acts—undermine mental and physical well-being and can create psychological burdens that dampen the protective effects of self-efficacy, income, and education. For example, high-achieving Black professionals may face constant scrutiny and biased judgments in their workplaces, which heightens stress and erodes the mental health advantages typically associated with socioeconomic success. This continuous exposure to interpersonal discrimination imposes an additional psychological tax on minoritized populations, diminishing the health benefits they might otherwise derive from their achievements and contributing to persistent health disparities across socioeconomic levels.

### A2. Social Stratification and Segregation

Social stratification and segregation are powerful forces that perpetuate racial and economic disparities, contributing to MDRs by limiting access to resources and opportunities in segregated communities [126]. Residential segregation, for example, often means that even affluent Black families live in neighborhoods with fewer amenities, lower-quality schools, and limited access to healthcare compared to similar-income White families [128]. This geographical divide reinforces social stratification, as minoritized communities are systematically excluded from the infrastructure and services that enhance well-being [129]. As a result, the potential health and economic benefits of higher SES are restricted by the social and spatial isolation of these communities, trapping them in a cycle where achievement does not yield comparable rewards and disparities continue to persist [130].

### A3. Lower Quality of Education

Educational quality remains unequal, often reflecting systemic racial and economic divides that leave minoritized students at a disadvantage, even when they attain higher levels of formal education [131]. Schools in predominantly Black or low-income neighborhoods frequently lack adequate funding, experienced teachers, and advanced academic programs [132]. Consequently, Black students often receive an education that, though it may formally fulfill requirements, does not equip them with the same opportunities or networks as their White counterparts [133]. This educational disparity limits the returns on educational attainment for minoritized individuals, as the quality of their education may not fully prepare them for competitive positions in the labor market or for further educational opportunities [134]. The result is an educational system that continues to uphold inequities, reducing the expected benefits of academic achievement and reinforcing the barriers faced by minoritized populations [135].

### A4. Labor Market Discrimination

Labor market discrimination is a critical mechanism that hinders the economic returns of education and experience for minoritized individuals. Black professionals, even with high levels of education and skill, often face hiring biases, wage disparities, and limited opportunities for advancement compared to their White peers. Research consistently shows that equally qualified Black job applicants are less likely to be hired, and when employed, they are often paid less and offered fewer leadership opportunities [59,80]. This discrimination in the workforce means that even high-SES Black individuals do not experience the same financial and occupational stability as similarly educated White individuals. The persistent bias within the labor market reduces the potential mental health and economic benefits of employment and professional success for Black individuals, perpetuating disparities and diminishing the returns on their hard-earned achievements.

### A5. Accumulation of Disadvantage from Childhood

The accumulation of disadvantages from an early age is another mechanism that shapes MDRs, as socioeconomic hardships and racial biases intersect throughout the life course to restrict opportunities for minoritized individuals [136]. Black children, for example, are often exposed to higher rates of poverty, poorer-quality education, and reduced access to healthcare, which creates a foundation of disadvantage that persists into adulthood [137]. As these children grow, the compounded effects of early hardships limit the health, educational, and economic benefits they may later gain from achieving high SES [138]. The impact of cumulative disadvantage suggests that even if individuals attain higher education or income later in life, the residual effects of early life adversities—such as chronic stress and limited social capital—dampen the positive outcomes typically associated with socioeconomic success, creating a lifelong cycle of diminished returns [139].

### A6. Internalized Racism

Internalized racism [81], wherein individuals begin to internalize negative societal beliefs about their own racial or ethnic group, is another mechanism that can weaken the benefits of self-efficacy and social achievements for minoritized populations [140]. The pervasive societal stigma against minoritized groups can lead individuals to doubt their worth, suppress their aspirations, or feel undeserving of their accomplishments [141]. This self-doubt, rooted in internalized racism, can reduce the psychological benefits that typically accompany high self-efficacy and economic stability. For instance, high-achieving Black individuals who have internalized racial stereotypes may experience imposter syndrome or fear of inadequacy in professional settings, which can increase stress and diminish mental health [142]. Internalized racism thereby limits the mental and emotional rewards that might otherwise stem from personal success, undermining the overall well-being of minoritized individuals despite their accomplishments [143].

### A7. Structural Racism in Banking, Policing, Real Estate, and Mortgage Lending

Structural racism is pervasive across various sectors, including banking, policing, real estate, and mortgage lending, and serves as a powerful mechanism of MDRs by limiting access to essential resources and security for minoritized populations [144]. In banking, for example, Black and other minoritized individuals are often denied loans or charged higher interest rates, which restricts their ability to build wealth and invest in high-quality housing [145]. In the realm of policing, over-surveillance of Black communities leads to heightened stress and fear, further diminishing the quality of life for these individuals. Additionally, discriminatory practices in real estate and mortgage lending make it more challenging for minoritized families to buy homes in desirable neighborhoods, reinforcing residential segregation and reducing property value gains [146]. These forms of structural racism systematically strip away the economic and social benefits that high SES could provide, exacerbating health disparities and ensuring that even financially successful Black individuals cannot fully enjoy the privileges of their status.

### A8. Legacy of Slavery and Jim Crow Laws

The legacy of slavery and Jim Crow laws [147] continues to cast a long shadow over the lives of Black Americans, as the effects of these historical injustices permeate every aspect of socioeconomic achievement. Enslavement and legalized segregation entrenched a racial hierarchy that not only limited the wealth and opportunities of Black individuals but also created enduring inequalities that affect subsequent generations [148]. The intergenerational trauma and economic disadvantage resulting from slavery and Jim Crow laws mean that Black families, on average, have less generational wealth, limited access to quality education, and a reduced ability to accrue assets compared to White families [149]. This historical legacy reinforces the MDRs framework, as Black individuals, despite their achievements, still grapple with a cumulative disadvantage that hinders their ability to secure health and well-being at the same level as their White peers [150]. The enduring impact of slavery and segregation underscores that addressing health disparities requires acknowledgment of historical injustices and the development of policies that actively work to dismantle their lingering effects.

## B. Mechanisms (mediators)

### B1. Lower Marriage Rates or Less Resources in Marriage

Lower marriage rates among minoritized populations, particularly Black Americans, act as a mediating factor in the diminished returns on socioeconomic status [82–84]. Marriage is often associated with economic and social benefits, including financial stability, shared resources, and emotional support, which collectively contribute to improved health outcomes. However, structural factors such as mass incarceration, economic instability, and relationship strain within minoritized communities have contributed to lower marriage rates. As a result, minoritized individuals may experience fewer of the economic and health benefits that marriage can provide, contributing to weaker health outcomes compared to their White counterparts with similar socioeconomic achievements.

### B2. Chronic Stress Across Domains

Stress across multiple life domains—work, family, financial, and social—serves as a significant mediator that diminishes the health benefits associated with high SES for minoritized individuals [85,86]. Racialized stressors, such as discrimination, microaggressions, and systemic biases, intensify the everyday stress load, which can have cumulative adverse effects on both physical and mental health. Chronic stress activates biological pathways that increase inflammation, disrupt metabolic function, and contribute to various chronic conditions. This prolonged stress exposure reduces the protective effects of income, education, and other socioeconomic resources, ultimately undermining well-being and accelerating health disparities for minoritized populations.

### B3. Environmental Toxins

Exposure to environmental toxins [116], including pollutants, lead, and other hazardous substances, is disproportionately high in communities where many minoritized populations reside [119,120]. This exposure often begins in early childhood and continues throughout life, contributing to respiratory issues, cardiovascular disease, and even cognitive impairments [151]. For individuals from minoritized backgrounds, living in toxin-heavy environments can nullify the health benefits typically associated with higher SES. Regardless of income or education, those in these neighborhoods are at heightened risk of toxin-related health issues, which undermines the expected health returns from socioeconomic achievements [52,153].

### B4. Health-Related Behaviors

Health-related behaviors, including substance use, smoking, and violence, can act as mediators that reduce the positive impact of socioeconomic status on health for minoritized populations [42,44]. For instance, structural inequalities and limited access to mental health services may lead to coping behaviors like smoking or substance use, which counteract the expected benefits of high SES [42,44]. Additionally, exposure to community violence can contribute to chronic stress, mental health challenges, and risky behaviors. These behaviors are often responses to environmental stressors rather than individual choices, and they significantly diminish the health benefits that high SES might provide, particularly for marginalized groups.

### B5. Living in Poor Neighborhoods

Even high-SES Black and other minoritized individuals are more likely to reside in poorer neighborhoods with limited access to healthcare, quality schools, and safe recreational spaces [117,118]. These neighborhood characteristics are critical determinants of health, shaping both physical and mental well-being. Poor neighborhoods often have fewer resources and higher crime rates, which can increase stress, limit access to preventive care, and reduce the likelihood of engaging in health-promoting activities [121]. As a result, even individuals with high socioeconomic status cannot fully benefit from their achievements if their neighborhood context limits their access to supportive resources, thus mediating the MDRs observed in health and well-being.

### B6. Intergenerational Mechanisms

Intergenerational transmission of disadvantage plays a crucial mediating role in MDRs, as the cumulative effects of historical and structural inequalities impact successive generations [25,87–89]. Economic hardships, health disparities, and exposure to discrimination often persist across generations, limiting the potential for socioeconomic gains to translate into health benefits. For instance, minoritized individuals may inherit economic disadvantage, lower educational opportunities, or poorer health from previous generations. This intergenerational cycle means that even high-SES individuals within these communities face challenges that inhibit the full realization of their socioeconomic achievements, perpetuating disparities across family lines.

### B7. Neurocognitive Mechanisms

Neurocognitive mechanisms, such as stress-related cognitive impairments and deficits in executive functioning, can mediate the relationship between SES and health outcomes for minoritized groups [154]. Chronic exposure to stress and discrimination can lead to neurocognitive challenges, including impairments in memory, attention, and problem-solving skills [155]. These cognitive effects may further limit the benefits of high SES. Additionally, neurocognitive impairment can increase vulnerability to mental health issues, reinforcing the diminished returns that often accompany socioeconomic achievements for marginalized populations. This evidence has recently been gathered from the ABCD study [28,88,90–93].

### B8. Impulsivity and Emotion Regulation

Impulsivity and difficulties with emotion regulation are important mediators that contribute to the weaker health benefits associated with SES for minoritized groups [94,95]. Chronic stress, discrimination, and adverse childhood experiences can all negatively impact emotional regulation skills, leading to greater impulsivity and difficulty managing stress in adulthood. Poor emotion regulation increases vulnerability to mental health issues, substance use, and other health-compromising behaviors, all of which counteract the protective effects of high SES. This pathway suggests that without addressing emotional and behavioral challenges, the benefits of socioeconomic success may remain limited for marginalized individuals [96–99].

### B9. Epigenetic Risks Across the Lifecourse

Epigenetic changes, which differ from genetic inheritance, involve the ways environmental exposures and stressors can alter gene expression over time. For minoritized individuals, chronic exposure to stress, discrimination, and environmental toxins can induce epigenetic changes that increase vulnerability to various health conditions, such as cardiovascular disease, diabetes, and mental health disorders [100]. Unlike genetic factors, epigenetic modifications are directly influenced by life experiences and can accumulate across the life course. This epigenetic risk mediates the relationship between SES and health outcomes, making it harder for high-SES minoritized individuals to fully benefit from their socioeconomic status due to the biological toll of lifelong adversity [122–125].

### B10. Allostatic Load

Allostatic load, or the cumulative wear and tear on the body from chronic stress exposure, is a powerful mediator that weakens the benefits of high SES on health outcomes for marginalized groups [101]. Continuous activation of the body’s stress response system can lead to physiological dysregulation, affecting cardiovascular, immune, and metabolic functions. Minoritized individuals, who often experience higher levels of stress across multiple domains, are more likely to have elevated allostatic load, even when they attain high SES. This biological burden diminishes the protective effects of socioeconomic success on health, making it a crucial mediator in understanding why minoritized populations face persistent health disparities despite their achievements.

## C. Policy Solutions, Recommendations, and Implications

### C1. Affirmative Action as a Mechanism for Equity:

Affirmative action policies remain vital for counterbalancing the structural barriers that continue to disadvantage minoritized groups. By providing targeted opportunities in education and employment, affirmative action helps level the playing field for those who have historically been marginalized [102]. These policies are not about giving unfair advantage; rather, they are about creating fair access to resources and opportunities for all individuals, particularly those who face systemic obstacles. By actively prioritizing minoritized individuals, affirmative action supports a fairer distribution of resources, which can foster more equitable health, educational, and economic outcomes.

### C2. Acknowledging the Struggles of the Non-White Middle Class:

Policy solutions may recognize the unique challenges faced by middle-class and high-achieving non-White individuals. While they have achieved economic or educational status, they often continue to experience discrimination and diminished returns on their accomplishments. Policies that address the specific needs of these individuals—who may face both racial biases and the pressures of maintaining their achievements in predominantly White spaces—are essential. Recognizing and supporting this group can help alleviate the psychological and economic strain they endure, which is often unrecognized in traditional policy frameworks focused solely on low-income populations.

### C3. More Comprehensive Policies:

Policies may include efforts to address disparities with more comprehensive approach, acknowledging that the complex effects of historical and structural racism cannot be fully addressed with a one-size-fits-all solution. Substantial progress may take time and effort, it needs more resources into minority populations to address these disparities effectively [103]. This approach recognizes that policies often produce different outcomes across racial and socioeconomic groups and that policies may be adaptable to meet evolving needs.

### C4. Recognizing the Long Timeline for Change:

Policies aimed at reducing disparities may be developed with the understanding that meaningful change may take decades or longer to achieve [104]. While history cannot be undone, its effects continue to shape present inequalities, and therefore, solutions need to be sustained and long-term. Policymakers should communicate openly that while progress will be gradual, persistent, well-supported policies can lead to substantial improvements over time. Accepting that disparities may persist while solutions are implemented encourages a long-term commitment to equity without unrealistic expectations of immediate results.

### C5. Avoiding Blame on Individual Differences:

It is crucial for policymakers to avoid attributing racial and economic disparities to individual shortcomings, such as perceived laziness, lack of ambition, or personality traits. Such explanations overlook the significant role of structural inequities and unfairly place the burden of disparities on those most affected by them. Effective policy solutions should instead focus on addressing the external barriers that prevent individuals from accessing the same opportunities as others, fostering an approach that is empathetic and rooted in an understanding of social context.

### C7. Developing Multi-Sectoral Policies:

Addressing disparities effectively requires multi-sectoral [105] approaches that engage various areas such as healthcare, education, employment, housing, and criminal justice. Coordinated policies across sectors ensure that improvements in one domain, such as education, are not undermined by barriers in another, such as labor market discrimination. A holistic, multi-sectoral strategy provides a more comprehensive framework for addressing the complex and interwoven issues that perpetuate racial and socioeconomic disparities.

### C8. Instilling Hope and Communicating Support:

Finally, policymakers should actively work to instill hope among minoritized populations, emphasizing that efforts to address disparities are ongoing and that their struggles are recognized at the highest levels [106,107]. This message is essential not to discourage individuals but to let them know that their challenges are seen, understood, and being addressed through meaningful policy changes. By fostering hope, policymakers can inspire resilience and reinforce the idea that while disparities exist, efforts are underway to create a fairer, more inclusive society where everyone has the chance to succeed.

## Conclusion

The enduring reality that “one must be twice as good to get half” encapsulates the challenges that high-SES Black individuals face in translating socioeconomic success into well-being and health benefits. Despite significant achievements in education, income, and self-efficacy, these groups experience weaker protective effects against health risks and adversity due to systemic barriers. Minorities’ Diminished Returns (MDRs) theory illuminates these disparities, attributing them to structural factors, including interpersonal discrimination, social stratification, labor market biases, and historical legacies of exclusion. The MDRs framework shows that the impact of income and education is mediated and weakened by chronic stress, environmental exposures, and the physical toll of discrimination over the lifespan. To achieve genuine social, economic, and health equity, public, economic, and health policies may extend beyond merely increasing SES among marginalized populations. Comprehensive, multi-sectoral policy changes are needed to dismantle structural barriers across domains such as housing, healthcare, and the criminal justice system. Implementing policies that recognize the unique challenges facing the non-White middle class, practicing humility in policymaking, and ensuring sustained long-term commitments are crucial for reducing disparities. By acknowledging these structural realities and challenging the “bootstraps” narrative, policymakers can foster an environment where success translates equally into health and well-being for all. While the path to equity is complex and long-term, addressing these barriers is essential to creating a society where success is accessible and beneficial across racial and socioeconomic lines.
